# Detection of aortic rupture using post-mortem computed tomography and post-mortem computed tomography angiography by cardiac puncture

**DOI:** 10.1007/s00414-015-1171-9

**Published:** 2015-03-15

**Authors:** Shu Zhou, Lei Wan, Yu Shao, Chongliang Ying, Yahui Wang, Donghua Zou, Wentao Xia, Yijiu Chen

**Affiliations:** Shanghai Key Laboratory of Forensic Medicine, Ministry of Justice, Institute of Forensic Science, 1347 West Guangfu Road, Shanghai, 200063 China

**Keywords:** PMCT, PMCTA, Cardiac puncture, Aortic rupture, Traffic accident

## Abstract

Post-mortem computed tomography (PMCT) and post-mortem computed tomography angiography (PMCTA) are rapidly becoming effective and practical methods in forensic medicine. In this article, we introduce a PMCTA approach by cardiac puncture and its application in a specific forensic case. A 50-year-old female sanitation worker was found dead on a road. External examination of the body revealed scattered abrasions and contusions over the chest. Autopsy was refused by the family members, and the body was examined with PMCT and PMCTA by cardiac puncture. Sternal fracture and rib fractures were detected by PMCT and aortic rupture by PMCTA. The cause of death was hemorrhagic shock due to traumatic aortic rupture. In certain circumstances, the combination of PMCT and PMCTA is helpful for forensic pathologists to determine the cause of death in cases involving traumatic vascular injury.

## Introduction

Traumatic aortic rupture is common in traffic accidents, and it is one of the most important and lethal causes of death [1–6]. Post-mortem computed tomography (PMCT) is rapidly becoming an effective and practical method in forensic medicine. The advantage of digital images from CT lies in two- and three-dimensional (3D) documentation, detection of certain injuries, and in the possibility of reconstructing the events leading to accidents [7, 8]. CT is the gold standard for the diagnosis of traumatic aortic rupture [9, 10].

In general, PMCT has proved to be sensitive for the detection of gunshot wounds, mechanical asphyxia, mechanical injury, and drowning [10–17]. However, the accuracy of non-contrast enhanced PMCT is low for the diagnosis of vascular disease or trauma. Fortunately, the whole-body post-mortem computed tomography angiography (PMCTA) is now available. PMCTA was first investigated by Jackowski et al. [18]. It allowed better visualization of arteries than the non-contrast-enhanced PMCT and permitted the evaluation of stenosis and occlusions [19]. It was reported that the combination of PMCT and PMCTA is very helpful in diagnosing some fatal injuries [20–22].

Once if minimally invasive autopsy with whole-body angiography was to be implemented for routine coronial autopsies, the number of cadavers to be examined at centers would run into thousands, and such complex approaches under these circumstances may be impractical. Saunders et al. [23] proposed that targeted cardiac PMCT could help to overcome this problem. However, this method can be used only in the diagnosis of coronary artery diseases. Here, we introduce, for the first time, a PMCTA approach by cardiac puncture, and the combination of PMCT and PMCTA was carried to determine aortic rupture in a forensic case.

## Material and methods

### Case report

A 50-year-old female sanitation worker was found dead on a road. The police investigation found no witnesses. The site of the incident was not covered by camera monitoring. Under investigation, the victim was previously asymptomatic with an uneventful medical history.

**Fig. 1:**
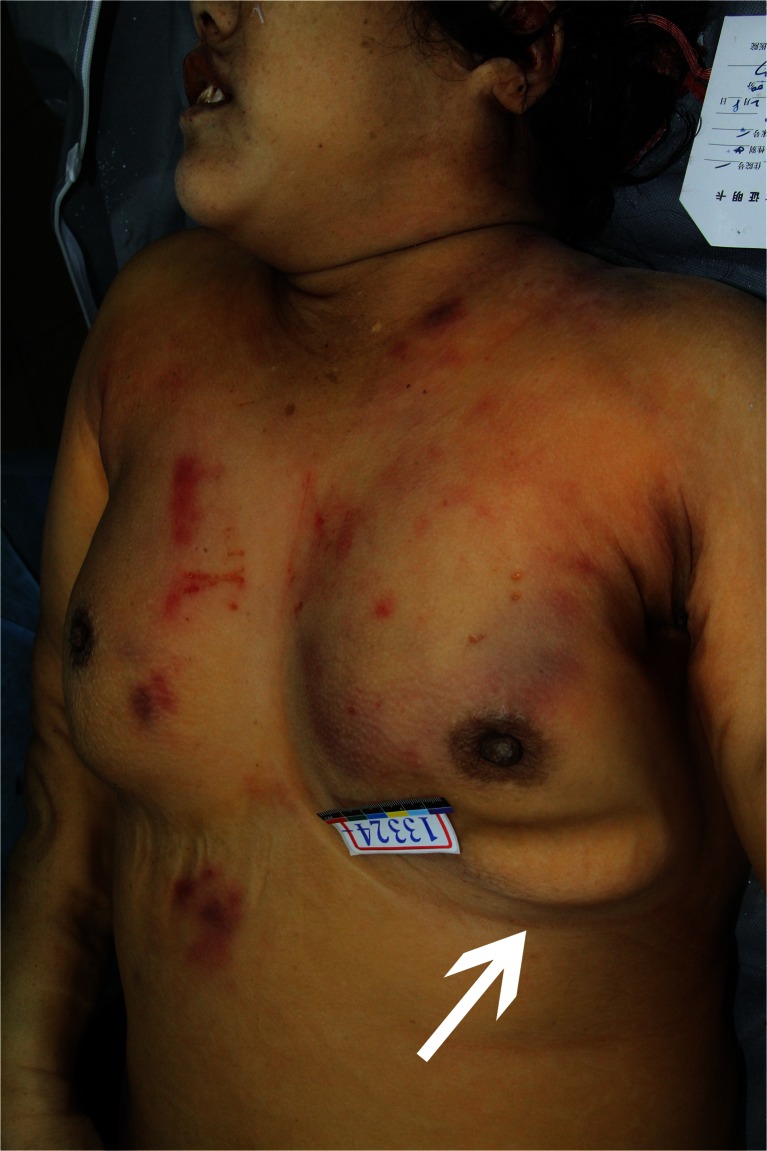
Scattered abrasions and contusions over the chest

The forensic examination of the body was proceeded 2 weeks after death. The body was of medium build. Scattered abrasions and contusions were observed over the torso and limbs, including both breasts, right lower abdomen and right inguinal region, back of the left hand, left knee, and medial side of the left thigh. No evidence of other recent injuries was detected (Fig. 1).

The cause of death and manner of the injury could not be determined by external examination. In China, autopsy was not enforced by law in traffic accident cases, and the family of the deceased did not want an autopsy to be carried out. Thus, external examination and examinations with minimal damage will be taken, instead.

### PMCT and PMCTA examination

PMCT was carried out 2 h after the external examination. The entire body was imaged using a 40-slice MSCT system (Definition AS; Siemens Medical Solutions, Munich, Germany). Acquisition of raw data was achieved using the following settings: voltage, 120 kV; current, 240 mA; and collimation, 6.0 × 1.0 mm. Image reconstruction was achieved at slice thicknesses of 5 and 0.625 mm, each with an increment of half the slice thickness-, soft tissue-, and bone-weighted reconstruction kernel. Image review and 3D reconstructions were carried out on a CT workstation (Syngo Imaging XS; Siemens Medical Solutions). Finally, the volume of hydrothorax was calculated automatically by the computer [24].

**Fig. 2:**
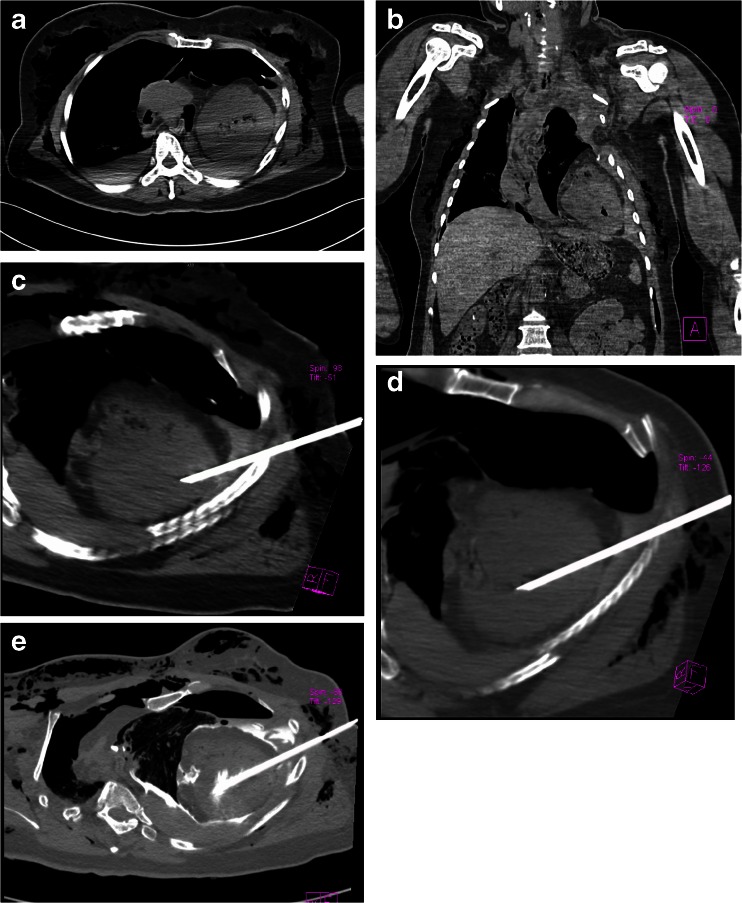
PMCTA procedure. **a**, **b** PMCT revealed fallen of the heart into the left costophrenic angle and pleural effusion. **c**, **d** A percutaneous puncture into the left ventricle through the intercostal area between the left fifth and sixth rib was conducted under CT guidance. **e** Administration of the contrast media

During PMCTA, a clinically ACN III^™^ biopsy core needle of 14 gauge × 160 mm was used. According to the position of heart viewing from multislice computed tomography (MSCT) images, the entry point and the path of the puncture were chosen towards the left ventricle (Fig. 2a, b). Then, a percutaneous puncture into the left ventricle through the intercostal area between the left fifth and sixth rib was conducted under CT guidance. The entire puncture depth was about 10 cm. During the puncture process, CT scanning was performed several times to ensure the needle in the right path (Fig. 2c–e).

After collecting samples of heart tissue and blood for toxicological analyses, 50 mL contrast media [meglumine diatrizoate and normal saline (0.9 %) at 10:1 ratio] was injected at the rate of 50 mL/8 s manually. Computed tomography angiography was carried out directly after administration of the contrast media solution.

## Results

**Fig. 3:**
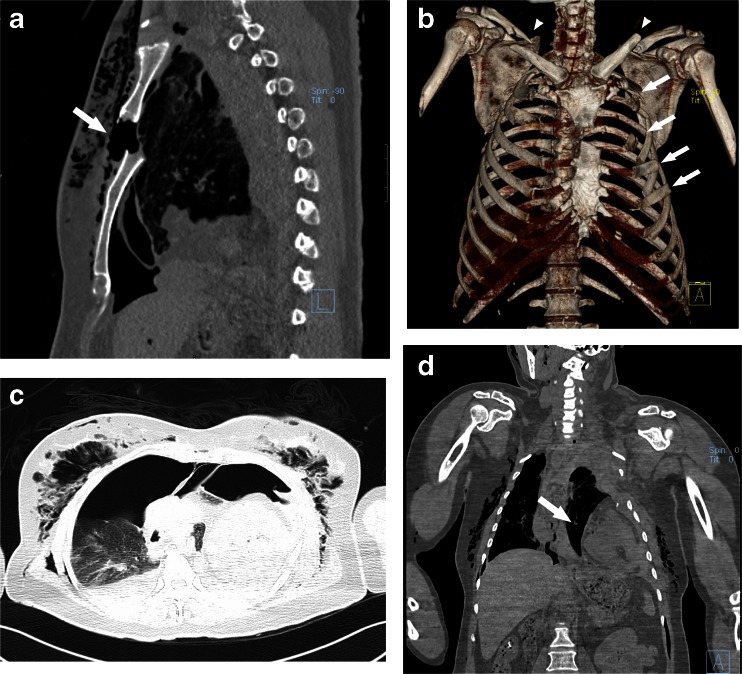
PMCT findings. **a**, **b** Sternal fracture with severely collapsed, multiple fractures of the bilateral clavicles and rib cages. **c**, **d** Bilateral hemopneumothorax, fallen of the heart into the left costophrenic angle and flattening of thoracic aorta

During PMCT, cutaneous emphysema of the chest wall was revealed. Sternal fracture with severely collapsed, multiple fractures of the bilateral clavicles and rib cage were detected. Bilateral pneumothorax and fallen of the heart into the left costophrenic angle were also observed. Flattening of thoracic aorta suggested hypovolemia (Fig. 3a–d). The stratification and the CT value of the pleural effusion suggested that there was also bilateral hematothorax. Calculated by CT device-related software, the volume of hematothorax was approximately 672.56 mL. No other injuries were detected.

**Fig. 4:**
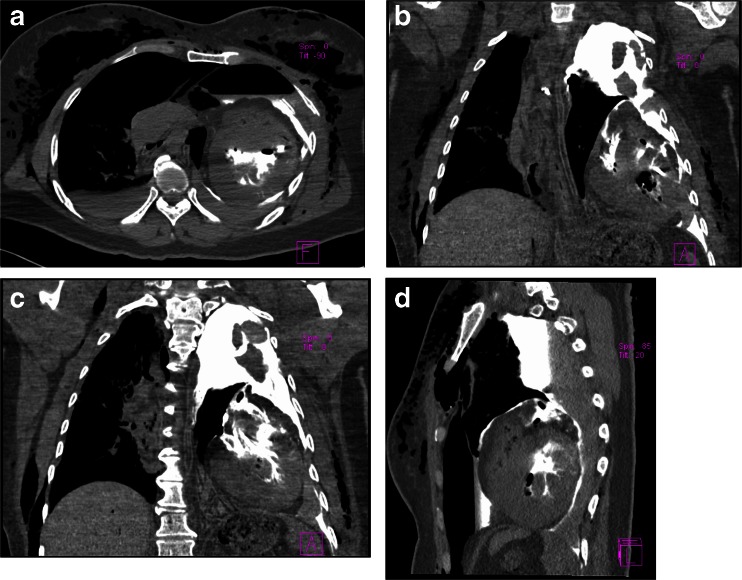
PMCTA findings. **a–d** Considerable leakage of contrast media into the left thorax was revealed. Only a small amount of contrast media entered the ascending aorta

During PMCTA, considerable leakage of contrast media into the left thorax was revealed (Fig. 4). Only a small amount of contrast media entered the ascending aorta, and no contrast effusion was found in the mediastinum or the pericardium. No evidence of rupture of the ventricle wall was found. Although the exact location and extent of the rupture could not be confirmed without autopsy, combining the findings of PMCT and PMCTA, we believed that there was a very large rupture between the aortic root and the ascending aorta, outside the pericardium, and might probably be a total transection.

Post-mortem drug screening in blood from the heart was negative for commonly used drugs. The test for alcohol was also negative.

According to external examination of the body and the findings of PMCT and PMCTA, the cause of death was concluded to mainly be the hemorrhagic shock due to traumatic aortic rupture, probably after the chest was being rolled over by wheels of the vehicle. Bilateral pneumothorax may also be included in the cause of death as well.

## Discussion

Worldwide, more than 1 million people are killed in road accidents each year. Hence, injuries sustained in motor-vehicle collisions are leading causes of death [25, 26], of which chest injuries account for a large part. Injury to the cardiovascular system resulting from blunt injuries to the chest comprises mainly rupture of the ventricles, atria, aorta, coronary arteries, and pericardium [27].

Medical imaging technology has been applied in forensic science for several years to detect injuries and determine the cause of death. PMCT and magnetic resonance imaging are promising modalities in forensic examinations and could be effective alternatives if an autopsy is refused or as a supplement to autopsy (e.g., to detect minor or occult injuries missed during autopsies) [28]. In our case, PMCT was used to detect fractures of the sternum, bilateral clavicles, and ribs, suggesting that the chest was compressed by blunt forces with a large contact area. The findings of PMCT also revealed signs of hypovolemia, suggesting potential cardiovascular injury.

PMCT has been applied in a few cases to detect cardiac and aortic injuries, including pericardial tamponade [1, 29, 30]. Combination of PMCT and PMCTA is time conserving and can be used to elicit 3D images of the blood vessels, which could enable the deep and narrow blood vessels to be revealed clearly.

Worldwide, there are three main forms of PMCTA, including the whole-body infusion angiographic techniques in Switzerland, the cardiopulmonary resuscitation to establish circulation in Japan, and the target coronary angiography techniques in UK. Differentiations between those PMCTA techniques depend upon the delivery, type, and the targets of the contrast agents. In addition, many studies [31] have focused on carrying out PMCTA of the heart or brain via catheters, which seems to have advantages. In our case, considering that the main injuries were probably in the aorta, carrying out PMCTA through cardiac puncture rather than through arteries in the lower limbs was more purposeful, effective, and time saving. PMCTA images revealed distinct evidence of aortic rupture in consequence. The advantages of our approach are that it is targeted, convenient, and time saving. However, it can be effective only in some specific cases, such as angiorrhexis. However, the quality of image is not comparable to the whole-body PMCTA images presented by some other groups. The main reason is considered as the differentiations in the device performance, scanning parameters, body condition, the performance of the device-dependent software and operators’ personal judgments through the digital post-processing, and was not much relevant to the approach itself.

According to the distribution and severity of the abrasions, contusions, bone fractures, as well as visceral and cardiovascular injuries, we believed that the chest sustained a huge blunt force, probably rolled over by the wheels of a motor vehicle, resulting in aortic rupture.

This fatal traffic accident was identified via PMCT and PMCTA. Sometimes, forensic experts could not determine the cause of death (e.g., aortic rupture) only by the injury patterns through external examination. In some specific cases, PMCT and PMCTA by cardiac puncture were time saving, convenient, effective, and, to some extent, of great value.
